# Multimodal imaging of a micro-anatomical structure in the vitreous base

**DOI:** 10.1186/s12886-023-03029-6

**Published:** 2023-06-20

**Authors:** Liang Han, Zhizhong Ma

**Affiliations:** Department of Ophthalmology, Peking University Third Hospital, Beijing Key Laboratory of Restoration of Damaged Ocular Nerve, 49 North Garden Road, Beijing, 100191 P. R China

**Keywords:** Vitreous base, Trauma, Ultrastructure, Transmission electron microscopy, Light microscopy

## Abstract

**Background:**

To describe an ultrastructure in the vitreous base (VB) and its micro-anatomical characteristics by multimodal imaging.

**Methods:**

Light and transmission electron microscopy of the VB were performed on specimens from post-trauma eyes and one healthy donor eye. Intra-operative fundus images associated with VB abnormalities were captured from 4 cases, including 2 retinal detachment (RD) with PVR eyes and 2 post-trauma eyes. Images during micro-anatomical observation of the three specimens were analyzed along with the fundus images obtained during vitrectomy.

**Results:**

Densely packed collagen fibers were observed by light microscopy between the pigment epithelium layer and uveal tissue within the ora serrata region in specimen 1 and the post-mortem healthy eye, respectively. A similar structure was also observed by transmission electron microscopy interior to the pigment epithelium layer and exposed to the vitreous cavity in specimen 2. The collagen fibers, which were termed ciliary body-choroid-retina (CB-C-R) connector, connects to the vitreous fibers interiorly, ciliary epithelium anteriorly, peripheral retina posteriorly, and uveal tissue exteriorly. The three different RD boundaries related to the posterior edge of the VB, ora serrata, and ciliary epithelium are demonstrated with the micro-anatomical characteristics of the CB-C-R connector.

**Conclusion:**

The CB-C-R connector exists deep in the VB.

## Key messages

The vitreous base (VB) is a transitional region connecting peripheral retina and ciliary epithelium layer. A structure named ciliary body-choroid-retina connector containing bundles of collagen fibers was discovered within the VB. Further investigation demonstrated its relationship with the surrounding tissues. The different boundaries of retinal detachment were related to the micro-anatomical characteristics of this structure.

## Background

The vitreous base (VB) is a region that extends approximately 1.5–2 mm anteriorly from the ora serrata (OS) on the pars plana and 3–5 mm posteriorly behind the OS on the retina; the posterior extension of the VB may enlarge with age [1–3]. The VB is involved in various vitreoretinal diseases [4–7]. However, some mechanisms in these diseases cannot be explained due to lack of the ultrastructure knowledge related to the VB.

**Fig. 1:**
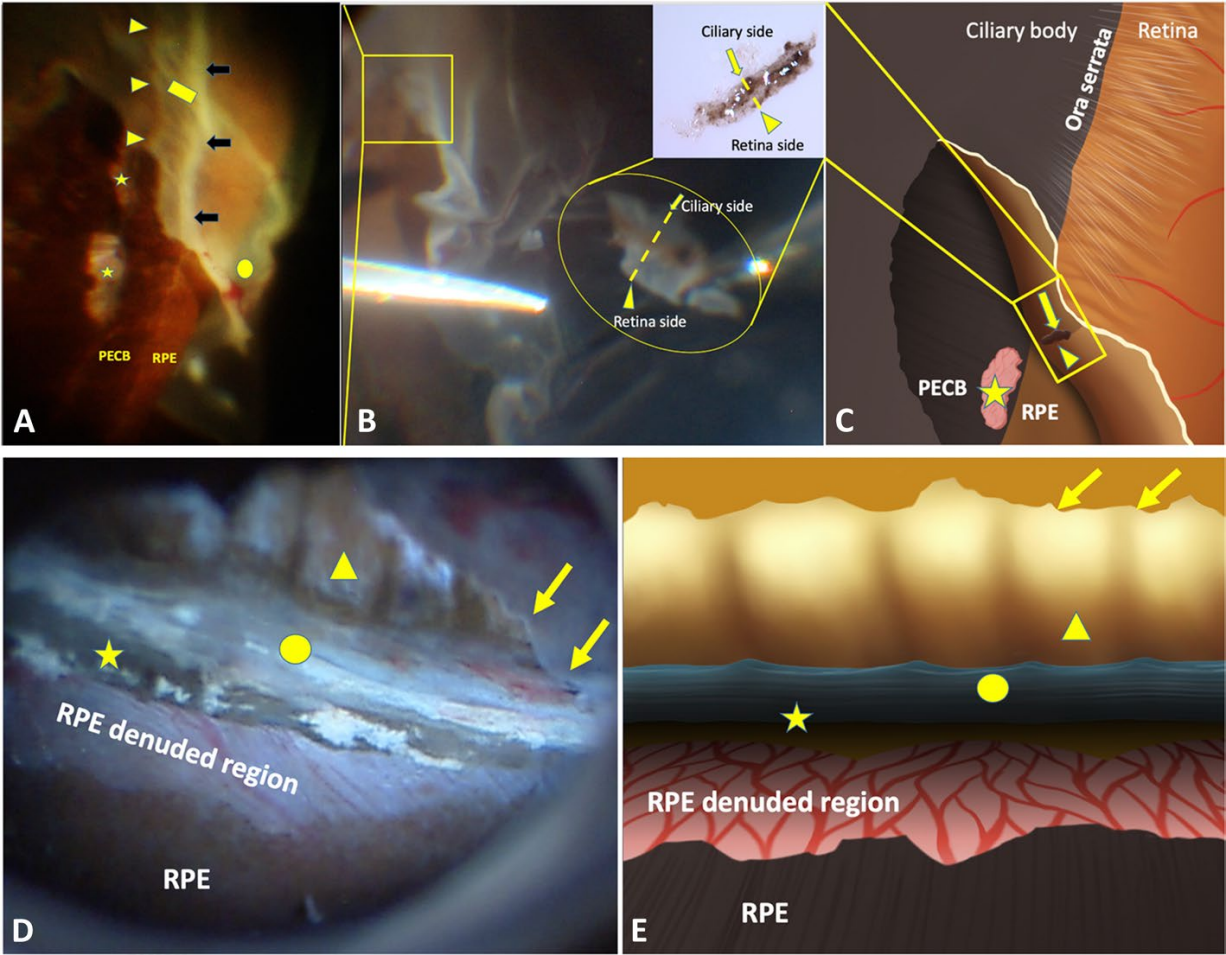
**A**: Patient 1. Blunt eyeball injury caused vitreous base (VB) avulsion, retinal detachment, ora serrata (OS) dialysis, and ciliary epithelium detachment. The VB region is consistent with the square region in Figure **B**. The OS border is marked by the arrows. The posterior edge of the VB (PEVB) is marked by the black arrows. The wrinkled retina between the OS border and PEVB located within the VB is marked by the yellow square. The pigment epithelium-denuded region exposed to the vitreous cavity is marked by the stars. **B**: The sample captured from the rectangular region shows that the pigment tissue torn from the region (star) in Figure A adhered to the OS. The intra-ocular view and gross appearance of the sample are showed in the circular and upright subsets, respectively. **C**: A diagram of Figure **A**. The star represents the same location of Figure A. The arrows and arrowheads are consistent with the those in Figure B. **D**: Patient 2. Direct view of the VB region in the eye that sustained contusion injury during vitrectomy. The avulsion of the ciliary epithelium is marked by the arrows. The pars plana (arrowhead) without the ciliary epithelium is exposed to the vitreous cavity. The uveal tissue (circle) is exposed to the vitreous cavity after severe VB avulsion. Choroidal vessels are observed through the retinal pigment epithelium-denuded region. **E**: A diagram of Figure **D** with corresponding markers

Clinically, progression of newly diagnosed rhegmatogenous retinal detachment (RD) ceases on the posterior edge of the VB (PEVB), whereas in traumatic cases or selected long-standing RD cases with proliferative vitreoretinopathy (PVR), OS and ciliary epithelium detachment (Fig. 1) may occur along with RD [8–10]. However, it remains unclear why some cases of RD do not continue beyond the PEVB or how the OS detaches from its underlying tissue. Meanwhile, we have also observed that torn pigment tissues from the underlying pars plana/choroidal substantia adhere firmly to the separated VB in blunt eyeball injuries with VB avulsion (Fig. 1B), and a whitish background in the pigment-denuded region with embedded choroidal vessels is often observed (Fig. 1D). It has been reported that the strong adhesion is caused by VB fibrils passing through the crypts of the peripheral retinal surface and blending with the lining basement membrane or with the plasmalemma of Müller cells [11]. However, current anatomical knowledge cannot clearly explain how large-scale pigmented sheet is torn from the underlying tissue.

Following observations of the VB micro-anatomical structure, we collected a series of peripheral retina images during vitrectomy surgery and compared these with images of ruptured VB tissue and healthy donor eyes obtained from microscopic observation. The ultrastructure located in the VB is fully visualized, and clinical VB abnormalities related to RD and trauma can be explained by the micro-anatomical characteristics of this structure. The findings of this study may help surgeons identify pathological signs and make rational decisions during surgery.

## Methods

**Fig. 2:**
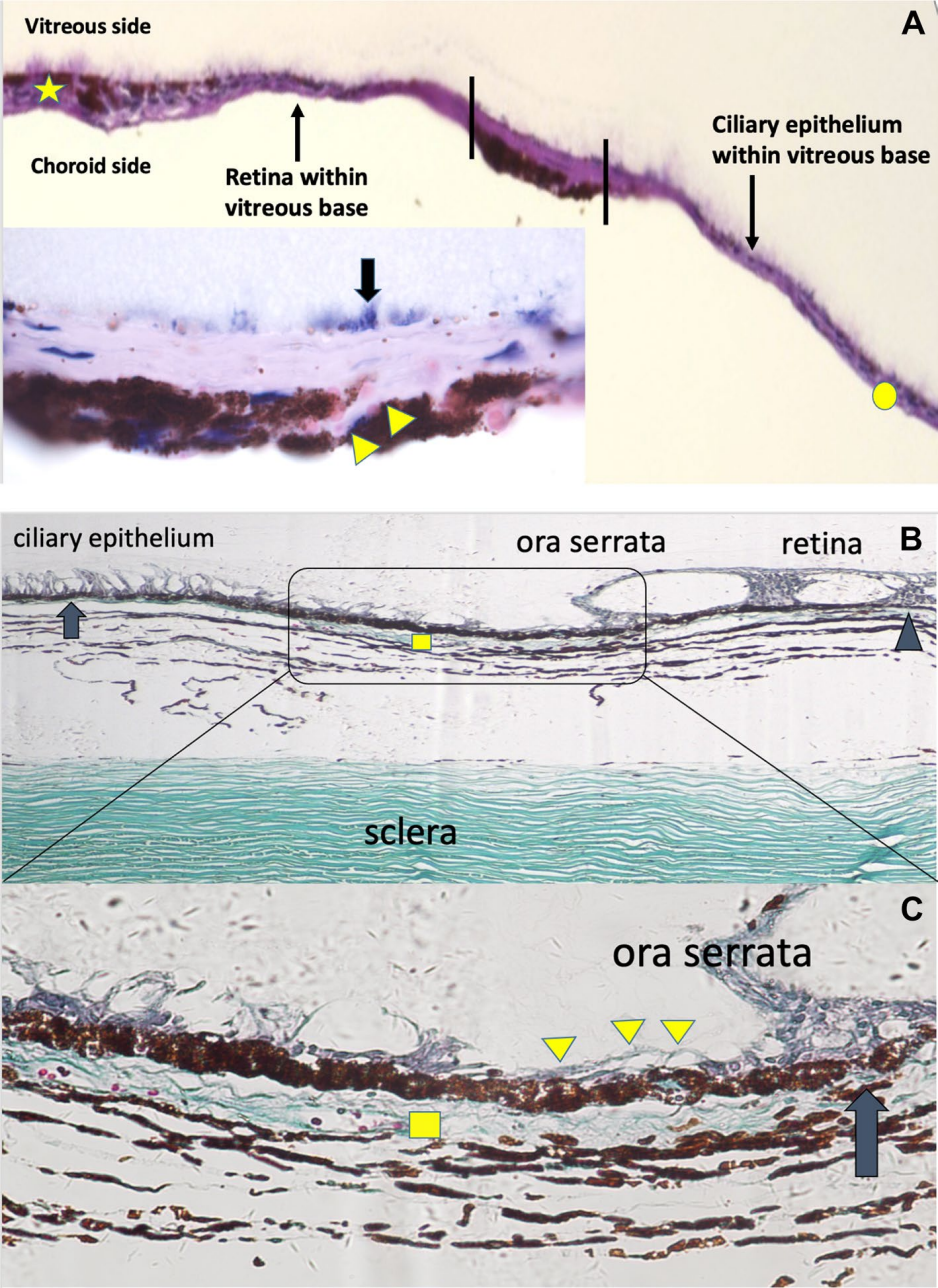
**A**: Periodic acid-Schiff staining of the specimen from patient 1. The spatial relationship is defined by the annotations. The circle is the epithelium of the ciliary body, while the star is the peripheral retina (× 20). The tissue within the vertical lines is from the ora serrata (OS) with torn pigment tissues, which is consistent with the appearance in the subset of Fig. 1B. The thick fiber bundles extend to the pars plana anteriorly and peripheral retina posteriorly. Magnification (× 100) of the tissue within the vertical lines is shown in the subset. It is mainly composed of bundles of dense collagen fibers without cellular structures rooted deeply into the underlying torn pigment tissues (choroidal melanocytes) by its branches (arrowheads). The vitreous fibers within the vitreous base (VB) region are directly connected to these fibers. B: VB region of a post-mortem healthy eye with Masson staining. The dense collagen fiber structures located between the pigment epithelium and choroidal tissue in the OS region (rectangular) (× 20). After staining, the structure appeared similar to the sclera, which is composed of collagen fibers. The ciliary pigment epithelium (arrow) shows a wider pigment band, while the retinal pigment epithelium layer located posterior to the OS appeared as a thin pigment layer (arrowhead). **C**: The collagen fibers (rectangular) arranged in multiple layers. The pigment epithelium is interrupted (arrow), and the underlying collagen fibers are connected to the tissues interior to the pigment epithelium layer through this break (arrow). (× 40) Non-cellular tissues interior to the pigment epithelium layer located between the peripheral retina and non-pigment epithelium (arrowheads)

This is a study of practical anatomy. The objects of the study are categorized into three groups: 1) Fundus images, which were obtained from 4 patients during vitrectomy surgery, included 2 with RD and 2 with blunt eyeball injuries; the images from 2 patients with blunt eyeball injuries were both taken from the avulsed VB region (Fig. 1A and D). The four fundus images were selected based on the corresponding anatomical characteristics of the VB, and all these four images were captured during the surgery through RESIGHT viewing system attached in OPMI LUMERA 700 Zeiss surgical microscope. The sclera depressing was introduced during surgery to expose the VB region clearly in all cases. 2) After locating the vitreous base abnormalities intraoperatively, light microscopic observations of vitreous base were applied on two specimens (Fig. 2A and B) from one eye (patient 1) post-trauma (Fig. 1A) and one healthy eye post-mortem, the purpose of the light microscopic observation is to know the cellular component of the VB specimens; and then 3) Transmission electron microscopic images of the specimen,which was captured from the other eye (patient 2) post-trauma during the vitrectomy surgery (Fig. 1D), were analyzed to understand the spatial relationship among different cells and tissues within the VB region.

**Fig. 3:**
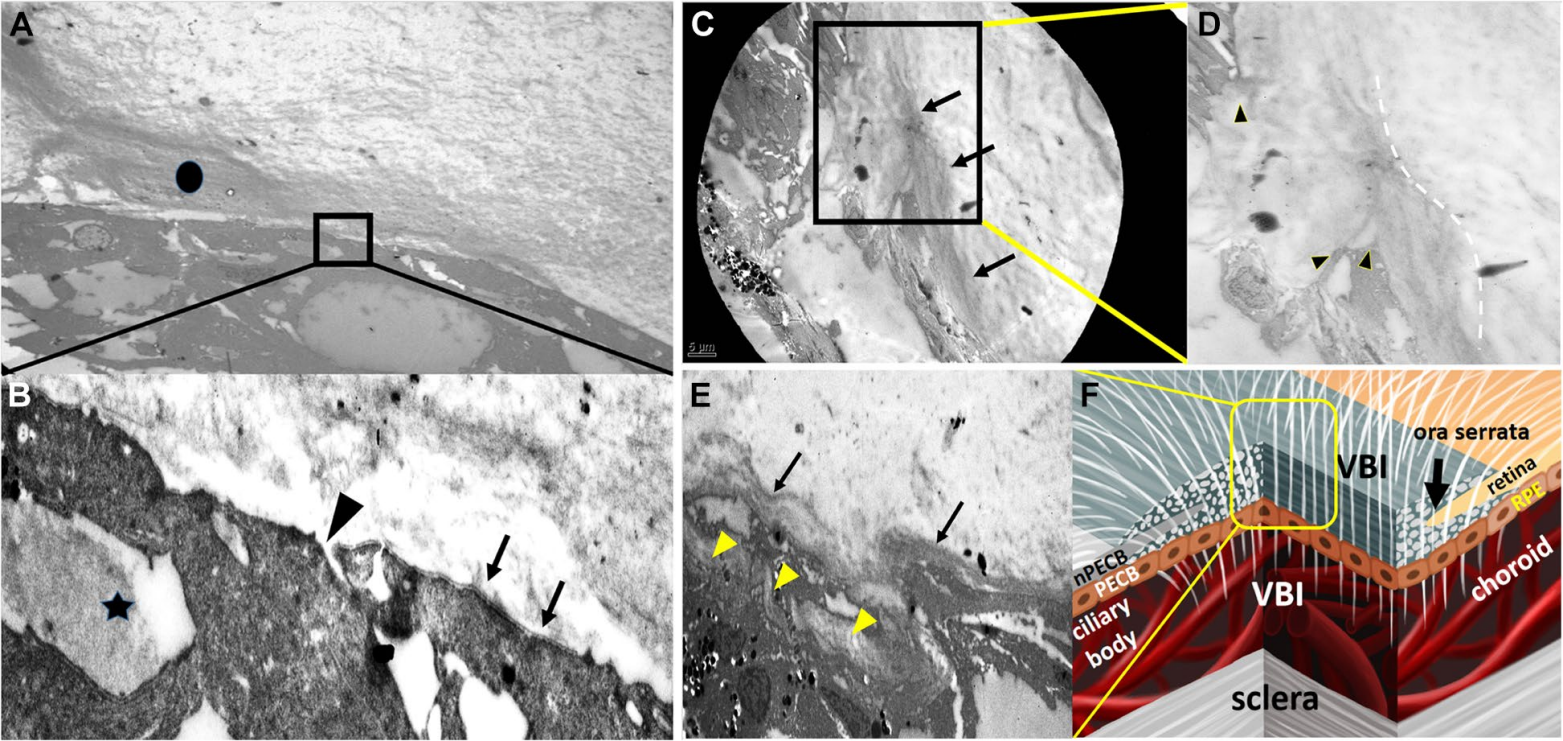
**A**: Vitreoretinal interface of specimen 2 showing the peripheral retina anterior to the posterior edge of the vitreous base (PEVB). The longitudinal bundles of thick collagen fibers (circle) are part of the VB insertion (VBI) structure, which gradually became thinner posteriorly (× 800). **B**: The internal limiting membrane (ILM) (arrows) is shown (× 6000). The interrupted ILM (arrowhead) in the region where the VBI is also located. The densely packed cross-sectional VBI collagen fibers are also observed within the retinal cells (star). **C**: Transmission electron microscopy of the vitreociliary interface of specimen 2. The densely packed chordal-like VBI collagen fibers insert into the non-pigment epithelium of the ciliary body (nPECB) (arrows, × 1,500). The pigment epithelium of the ciliary body located next to the nPECB. **D**: The border representing different fiber densities between the VBI and ordinary vitreous fibers are marked by the dashed line. The superior portion of the nPECB is pulled towards the vitreous cavity by the VBI (arrow heads, × 2,500). **E**: Transmission electron microscopy of the OS region of specimen 2 (× 2,500). The vitreous fibers are directly connected to the dense collagen fibers forming the VBI structure (arrows). The fiber bundles run into the pigment epithelium cells (arrow heads). F: The diagram shows the three-dimensional spatial relationship among the different layers. The content of the yellow square is consistent with the demonstration in Figure **F**, which involves the vitreous fibers interiorly, dense collagen fibers in the middle, and pigment epithelium exteriorly

Two samples containing detached ciliary epithelium and peripheral retina along with the avulsed VB region were both extracted from patient 1 and patient 2 during the surgery. The specimen from patient 1 was immediately placed on a cellulose acetate membrane during surgery (Fig. 1B subset) and embedded in 10% paraformalin for light microscopic observation under hematoxylin and eosin and periodic acid-Schiff staining (Fig. 2A). The specimen from patient 2 was embedded in 2% glutaraldehyde for transmission electron microscopic observation (Fig. 3). The post-mortem healthy eye was stained with Masson stain for microscopic observation (Figs. 2B and C).

The surgical microscope used for operation was OPMI LUMERA 700 ZEISS. The microscope for histopathology observation was Olympus BX53A with U-LHLEDC. The transmission electron microscope was JEM-1230,JEOL.

Before surgery, we had planned to obtain the samples containing detached ciliary epithelium and peripheral retina within the avulsed VB region caused by trauma if the detached tissue cannot be re-attached during the revised surgery for patient 1 and patient 2. The patients were notified of our plan before surgery, and consent was obtained. This study was approved by the Institutional Review Board of Peking University Third Hospital and was conducted in accordance with the tenets of the Declaration of Helsinki.

## Results

### Microscopic observations of the specimen from patient 1 and the post-mortem healthy eye

Patient 1 was diagnosed with traumatic RD associated with VB avulsion. The intermittent band-like pigment denuded regions (Fig. 1A stars) on the pigment epithelium layer were exposed to the vitreous cavity corresponded to OS dialysis (Fig. 1A). Pigment tissue was detected in the middle of the specimen with the detached retina and detached ciliary non-pigment epithelium on each side (Figs. 1B and C). The specimen from patient 1 was sectioned following the dashed line in the subset of Fig. 1B. The anterior ciliary epithelium, OS with pigment, and peripheral retina of the VB were all detected by light microscopy (Fig. 2A), whereas the pigment epithelium layer was absent. The OS region in the middle of the sample with the underlying choroidal melanocytes was the thickest part with bundles of dense collagen fibers above. Anteriorly, the ciliary epithelium was thin, while the peripheral retina appeared thicker posteriorly. The vitreous fibers (Fig. 2A arrows) above connected to bundles of dense collagen fibers with its branches (Fig. 2A subset, arrowheads) deeply inserting into the underlying choroidal tissues. The multiple connections among vitreous fibers, dense collagen fibers, and choroidal tissues form the anatomic basis of VB insertion (VBI).

By observing the OS region of the post-mortem healthy eye, a similar dense collagen fiber structure was also identified between the pigment epithelium layer and uveal melanocytes (Fig. 2B). Meanwhile, non-cellular tissue inside the pigment epithelium (Fig. 2C arrow heads) was observed, which was connected to the dense collagen fibers within the uvea through an interrupted pigment epithelium break (Fig. 2C arrow); this non-cellular tissue was directly exposed to the vitreous cavity.

### Transmission electron microscopic observation of the interface of the detached OS, non-pigment ciliary epithelium, and peripheral retina within the VB region in patient 2 (Fig. 3)

The VBI, which extended to the peripheral retina, was composed of thick and dense collagen fibers that became thinner posteriorly. An intermittent internal limiting membrane (ILM) was observed along with the VBI, and intra-cellular collagen fibers from the VBI were observed near the interrupted ILM (Fig. 3B). The wrinkled appearance of the detached retina within the VB region (Fig. 1A yellow square) was associated with close connection between the VBI and retina.

The VBI that extended towards the ciliary body was also composed of thick and dense collagen fibers, which pulled the non-pigment epithelium of the ciliary body (nPECB) into the vitreous cavity, causing separation between the PECB and nPECB (Figs. 3C and D).

The VBI in the OS region was composed of vitreous fibers interiorly, dense collagen fibers in the middle, and pigment epithelium layer exteriorly (Figs. 3E and F). Neither the peripheral retinal structure nor the non-pigment epithelium layer was detected interior to the pigment epithelium layer, which are consistent with those observed in the post-mortem healthy eye (Fig. 2C arrows).

### Micro-anatomical characteristics of the ciliary body-choroid-retina connector (CB-C-R)

**Fig. 4:**
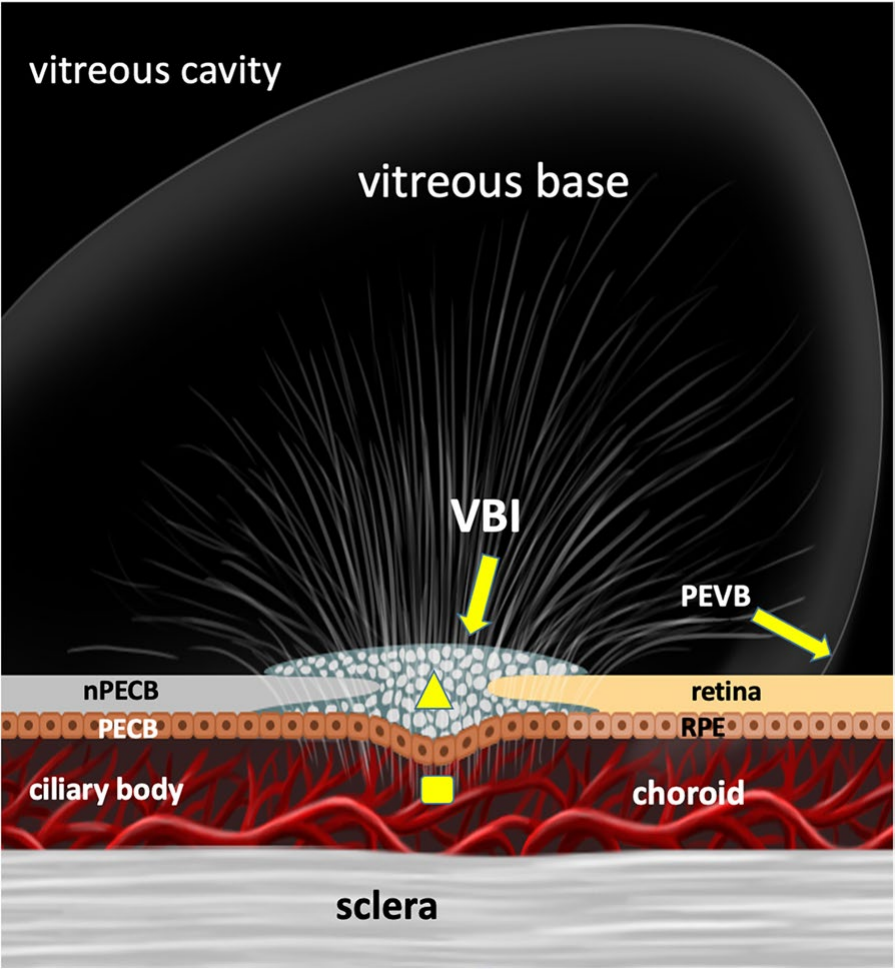
Bundles of dense collagen fibers (triangle) in the ora serrata (OS) connected to the various tissues around, which is called the ciliary body-choroid-retina connector. nPECB: non-pigment epithelium of ciliary body. VBI: vitreous base insertion

The collagen fibers (Fig. 2A arrowheads, Fig. 2C square, Fig. 3A circle, Fig. 3B star, Fig. 3C arrows, Fig. 3E arrows and arrowheads), which are collectively termed as the CB-C-R connector, connects to the vitreous fibers interiorly, ciliary epithelium anteriorly, peripheral retina posteriorly, and uveal tissue exteriorly (Fig. 4). The compromised CB-C-R connector in the two trauma cases resulted in the exposure of the uveal tissue to the vitreous cavity (Fig. 1A star and Fig. 1D circle). The densely packed chordal-like VBI collagen fibers (Fig. 3A circle, Fig. 3C arrows and Fig. 3E arrows), which are part of the CB-C-R connector structure, are much denser than ordinary vitreous fibers.

### The relationship between the VB anatomical characteristics and RD with three different boundaries

**Fig. 5:**
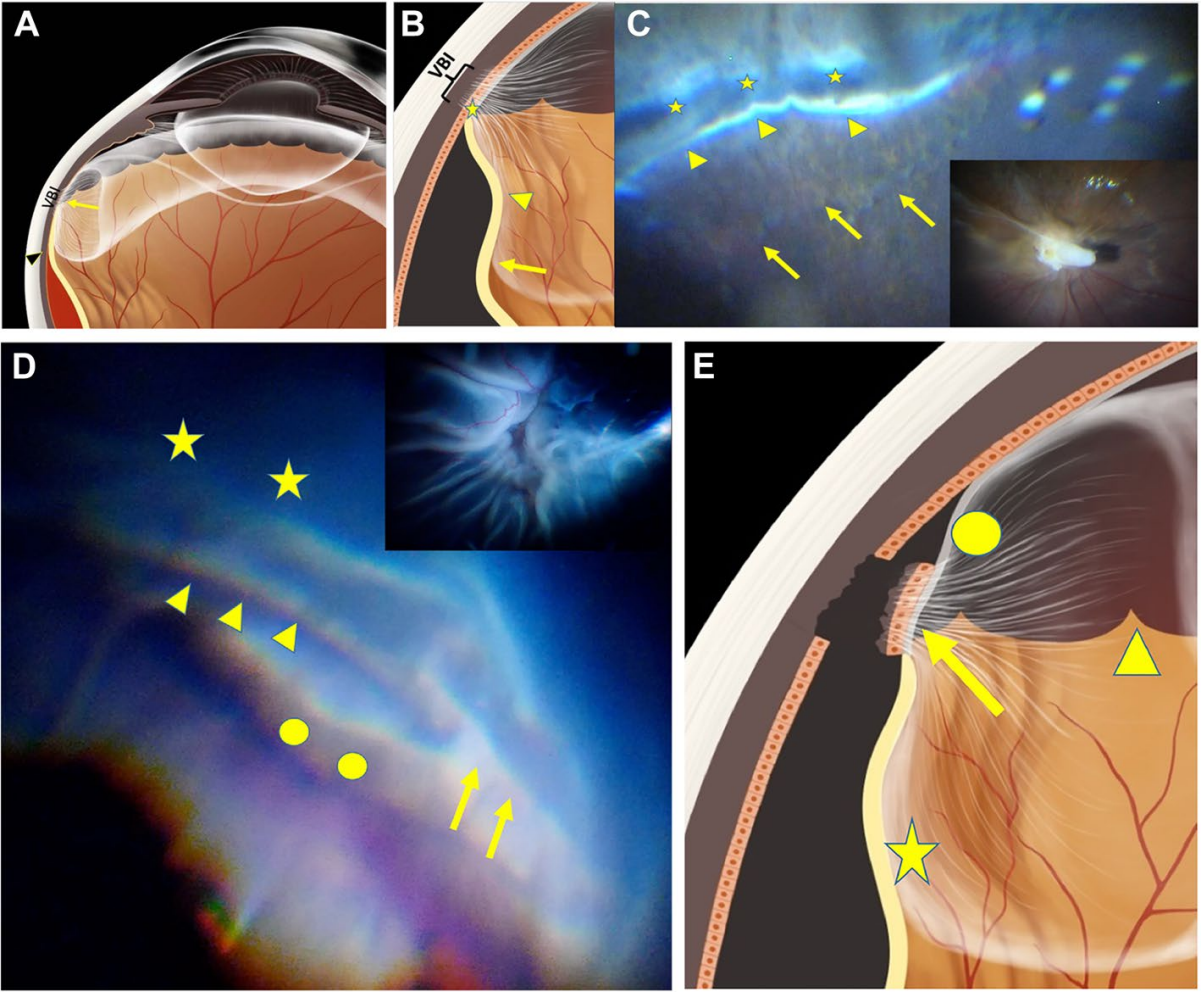
**A**: The diagram demonstrates retinal detachment ceasing at the posterior edge of the vitreous base (arrow heads). The red color indicates subretinal fluid with the detached retina above. **B**: The boundary of retinal detachment (RD) ceased posterior to the ora serrata (OS). The arrow points to the posterior edge of the vitreous base (PEVB), the star indicates the OS with the VB insertion (VBI). The arrowhead points to the RD within the VB. **C**: The subset shows a long-standing RD with advanced proliferative vitreoretinopathy. All markers have similar representations as those in Figure **B**. The light shown by arrowheads indicates the ridge of RD pushed by perflurocarbon. The region posterior to the PEVB (arrows) shows a different color compared to that anterior to the PEVB. D: RD extending beyond the OS. The arrowheads indicate the OS with no detachment, while the arrows indicate the detached OS. The circles represent the detached non-pigment epithelium of the ciliary body (nPECB) connected to the detached OS. The PECB is also detached in this region along with the detached nPECB. The stars indicate areas of RD. The subset shows advanced proliferative vitreoretinopathy. E: All markers have the same representations as those in Figure **D**

1. RD ceases at the border of the PEVB (Fig. 5A). Clinically, cases of rhegmatogenous RD usually cease at the PEVB. This can be explained by the close connection between the VBI and retinal pigment epithelium layer within the VB region, which prevents RD progression towards the OS. This connection is demonstrated by 1) dense collagen fibers of the VBI extending into the deeper pigment tissues (Fig. 2A arrow heads); and 2) the denuded pigment epithelium of the ciliary body (PECB) (Fig. 1A stars) along with the avulsed VB in patient 1.
2. RD extends beyond the PEVB and ceases posterior to the OS (Figs. 5B and 5C). RD in a silicone oil tamponade eye recurred with advanced PVR (Fig. 5C subset). After perfluoracarbon liquid was injected into the vitreous cavity, the subretinal fluid was pushed to the periphery, ceasing just posterior to the OS. (Figs. 5B and 5C stars). The VBI (Fig. 3F and Fig. 4) in the OS limited the RD. The wrinkled RD (Fig. 1A square) between the PEVB and OS was caused by VBI traction and its tight interaction with the peripheral retina (Fig. 3A circle and 3B star). The different color in the region posterior and anterior to PEVB (Fig. 4C) was due to the compromised retinal pigment epithelium layer anterior to the PEVB when the VB retina detached.
3. The RD extends beyond the OS, leading to ciliary epithelium detachment (Fig. 5D and E). This usually occurs in cases of long-term RD with advanced PVR and in trauma (Fig. 1). The tight connection between the CB-C-R and its underlying tissue is compromised by the traumatic force or long-standing traction of PVR, leading to the exposure of the uveal tissue to the vitreous cavity (Figs. 1D and 1E circle).

## Discussion

By combining the microscopic findings and fundus images obtained during vitrectomy surgery, we demonstrated the location of the CB-C-R connector in the VB, which is composed of dense collagen fibers that are different from vitreous fibers. The spatial relationship between the connector and its surrounding tissues were also defined (Fig. 4). The important anatomical markers related to the CB-C-R connector are the PEVB, VBI, and OS, which were all demonstrated clearly by both histological and clinical observations.

The reasons of choosing light microscopy and transmission electron microscopy are as follows: 1) It is a mandatory procedure to observe the samples obtained from patients during surgery for histopathological diagnosis. 2) The transmission electron microscopic observation can demonstrate clearly the cellular content of CB-C-R as well as their spatial relationship with adherent tissues in the VB region. 3) Due to the limited sample size and numbers of specimen, it would be hard to choose other modalities (immunohistochemistry staining or scanning electron microscopic observation) for histopathologic observation to demonstrate the structure of CB-C-R.

Ciliary epithelium tears and OS dialysis, which break the connection between the CB-C-R connector with its surrounding tissues including the choroid, OS, ciliary epithelium, and VB, may occur following VB avulsion. Feng [12] reported that in 10 ruptured eyeball injuries, choroidal rupture occurred posterior to the pars plana in all cases. The location of the CB-C-R connector was consistent with the locations of choroidal rupture, which may be explained by the CB-C-R connector being pulled away vertically from the choroid towards the vitreous cavity by the VBI and lead to choroidal rupture.

The different boundaries of RD are associated with the different compromised connections among different tissues within the VB. 1) RD ceases posterior to the PEVB (Fig. 5A). Histopathologically, the VBI anterior to the PEVB allows the VB to be firmly attached to the retina (Fig. 3A circle and 3B star). Meanwhile, the connection between the retina and the underlying retinal pigment epithelium layer is stronger in the anterior PEVB than in the posterior PEVB. Although we did not obtain images of the anatomical connection between the retina and retinal pigment epithelium layer in the VB, we observed that the retinal pigment epithelium sheet was attached to the detached retina, leaving a large retinal pigment epithelium-denuded region next to the OS, as shown in the fundus image of patient 2 (Fig. 1D). 2) RD extends beyond the PEVB and cease at the OS where the CB-C-R is located (Figs. 5D and 5E). As PVR progresses into the circumferential type (grade C) that leads to centrepedal-contracted RD, the VBI in the OS is the only site where vitreous fibers are connected to the pigment epithelium layer [13] (Fig. 5E). The CB-C-R connector functions as a fixed and firm plug that resists the pulling force of PVR. 3) In cases of trauma and long-standing RD with severe PVR, the RD perimeter extends beyond the OS and involves the ciliary epithelium (Figs. 1 and 5D). Clinically, OS avulsion, RD, and ciliary epithelium detachment were observed. Histopathologically, the CB-C-R connector separated from the underlying choroidal tissue and pigment/non-pigment epithelium along with RD, which exposed the uveal tissue to the vitreous cavity.

Although the techniques and instruments have been improved dramatically, circular scleral buckle procedure after vitrectomy still plays an important role in preventing retinal tears posterior to the PEVB in eyes undergoing long surgeries or multiple posterior vitreous detachment (PVD) [14–17]. Variations of the VBI, which may cause complications [3, 18], may also be prevented by circular scleral buckle surgery. When PVD occurs, the peripheral retina posterior to the PEVB is most vulnerable to the tangential pulling force from the VBI due to its connection to the vitreous fibers and peripheral retina. Scleral buckle procedures reduce vitreous traction to the retina and strengthens the stability of the transition zone between regions of PVD and non-PVD posterior to the PEVB.

The number of samples in this study was limited. Additionally, some samples were obtained from eyes post-trauma, which may not reflect normal VB status. Hence, any conclusions drawn from the study should be taken with caution.

## Data Availability

The data and material used or analysed during the current study are available from the corresponding author on reasonable request.
